# Long-term cold, freezing and drought: overlapping and specific regulatory mechanisms and signal transduction in tea plant (*Camellia sinensis* (L.) Kuntze)

**DOI:** 10.3389/fpls.2023.1145793

**Published:** 2023-05-10

**Authors:** Lidiia Samarina, Songbo Wang, Lyudmila Malyukova, Alexandr Bobrovskikh, Alexey Doroshkov, Natalia Koninskaya, Ruset Shkhalakhova, Alexandra Matskiv, Jaroslava Fedorina, Anastasia Fizikova, Karina Manakhova, Svetlana Loshkaryova, Tsiala Tutberidze, Alexey Ryndin, Elena Khlestkina

**Affiliations:** ^1^ Federal Research Centre the Subtropical Scientific Centre, Russian Academy of Sciences, Sochi, Russia; ^2^ Center of Genetics and Life Sciences, Sirius University of Science and Technology, Sirius, Russia; ^3^ Institute of Cytology and Genetics Siberian Branch, Russian Academy of Sciences, Novosibirsk, Russia; ^4^ Federal Research Center, N. I. Vavilov All-Russian Institute of Plant Genetic Resources (VIR), Saint Petersburg, Russia

**Keywords:** tea plant (*Camellia sinensis*), stress tolerance, cell wall biosynthesis, lipid metabolism, light perception, phenilpropanoid pathway, differentially expressed genes, coexpression analysis

## Abstract

**Introduction:**

Low temperatures and drought are two main environmental constraints reducing the yield and geographical distribution of horticultural crops worldwide. Understanding the genetic crosstalk between stress responses has potential importance for crop improvement.

**Methods:**

In this study, Illumina RNA-seq and Pac-Bio genome resequencing were used to annotate genes and analyze transcriptome dynamics in tea plants under long-term cold, freezing, and drought.

**Results:**

The highest number of differentially expressed genes (DEGs) was identified under long-term cold (7,896) and freezing (7,915), with 3,532 and 3,780 upregulated genes, respectively. The lowest number of DEGs was observed under 3-day drought (47) and 9-day drought (220), with five and 112 genes upregulated, respectively. The recovery after the cold had 6.5 times greater DEG numbers as compared to the drought recovery. Only 17.9% of cold-induced genes were upregulated by drought. In total, 1,492 transcription factor genes related to 57 families were identified. However, only 20 transcription factor genes were commonly upregulated by cold, freezing, and drought. Among the 232 common upregulated DEGs, most were related to signal transduction, cell wall remodeling, and lipid metabolism. Co-expression analysis and network reconstruction showed 19 genes with the highest co-expression connectivity: seven genes are related to cell wall remodeling (*GATL7*, *UXS4*, *PRP-F1*, *4CL*, *UEL-1*, *UDP-Arap*, and *TBL32*), four genes are related to calcium-signaling (*PXL1*, *Strap*, *CRT*, and *CIPK6*), three genes are related to photo-perception (*GIL1*, *CHUP1*, and *DnaJ11*), two genes are related to hormone signaling (*TTL3* and *GID1C-like*), two genes are involved in ROS signaling (*ERO1* and *CXE11*), and one gene is related to the phenylpropanoid pathway (*GALT6*).

**Discussion:**

Based on our results, several important overlapping mechanisms of long-term stress responses include cell wall remodeling through lignin biosynthesis, o-acetylation of polysaccharides, pectin biosynthesis and branching, and xyloglucan and arabinogalactan biosynthesis. This study provides new insight into long-term stress responses in woody crops, and a set of new target candidate genes were identified for molecular breeding aimed at tolerance to abiotic stresses.

## 1 Introduction

Low temperatures and drought can lead to decreased water potential of plant tissues and induce reactive oxygen species accumulation, which causes severe damage to various cellular components (Chaves et al., 2003; Minhas et al., 2017). Lately, significant progress has been made in the identification of stress-inducible genes and components of signaling pathways involved in a variety of abiotic stresses. Thousands of genes are involved in response to each stressor, and the relationships among genes remain largely unknown (Hao et al., 2018; Xia et al., 2019).

In nature, plants often face several abiotic stresses rather than a particular one at the same time (Ahuja et al., 2010). Cold, freezing, and drought induce common and specific sets of signaling pathways and regulatory mechanisms following biochemical responses affecting plant phenotype (Zhou et al., 2019). Consequently, cold tolerance also promotes drought tolerance in plants, which is consistent with an increase in osmo-regulatory compounds and antioxidant enzyme activities (Li et al., 2019). Thus, it is necessary to better understand the cross-talk genetic mechanisms for the development of cultivars that are tolerant to both environmental factors that can largely contribute to the enhancement of crop productivity under changing climates worldwide (Minhas et al., 2017). Additionally, comparison of the molecular profiles of an organism under different stresses would allow us to identify conserved stress mechanisms in woody crops (Amrine et al., 2015; Muthuramalingam et al., 2017; Chamani Mohasses et al., 2020).

The cross-talk transcriptomic responses between cold and drought have been reported in a few studies. Among them, much more DEGs were upregulated under cold conditions rather than drought in tea plants (Zheng et al., 2015), apples (Li et al., 2019), and maize (Lu et al., 2017). However, a significant number of DEGs were upregulated during drought as compared to cold in cassava (Li et al., 2017). In total, 56% of common genes expressed under drought and cold stresses were related to 43 transcription factor families (primarily *WRKY*, *NAC*, *MYB*, *AP2/ERF*, and *bZIP*) in Arabidopsis (Sharma et al., 2018). Many transcription factors and metabolite-related genes have been shown to be involved in both the cold and drought responses of tree species (Wang et al., 2012; Ban et al., 2017). ABRE-binding proteins and ABRE-binding factor TFs control gene expression in an ABA-dependent manner. *SNF1*-related protein kinases2, group A2C-type protein phosphatases, and ABA receptors were shown to control the ABA signaling pathway. ABA-independent signaling pathways such as *DREB* and *NAC* TFs are also involved in stress responses, including drought, heat, and cold (Nakashima et al., 2014; Pareek et al. 2017). In contrast to Arabidopsis (Matsui et al., 2008; Sham et al., 2014), maize (Shan et al., 2013), and rice (Rabbani et al., 2003). In woody crops, little has been reported about transcriptome dynamics and crosstalk responses to drought, cold, and freezing. Moreover, available data were obtained for short-term stress treatments, but long-term stress and recovery were not sufficiently studied.

Among tree crops, the tea plant (*Camellia sinensis* L.) is one of the most important commercial crops in China, India, Sri Lanka, Kenya, and certain Caucasian and Middle Eastern countries (Turkey, Georgia, Russia, and Azerbaijan). This perennial evergreen crop is grown in more than 60 countries on five continents, from 49°N in Ukraine to 33°S in South Africa (Turkozu and Sanlier, 2017). In the most tea-producing countries, plantations are affected by drought and cold stress, which significantly reduce the yield and decrease the distribution of the crop in colder areas. The available studies on tea plants confirmed that the key cold regulators *ICE*, *CBF*, and *DHN* are related to an ABA-independent responsive pathway and participate in both cold and drought stress and in other abiotic stress responses (Liu et al., 2015; Singh, and Laxmi 2015; Ban et al., 2017; Hu et al., 2020). In addition, several transcription factor families (*AP2/EREBP*, *WRKY*, *bHLH*, *NAC*, *MYB*, *HSP*, *LEA*, *CML*, *bZIP*, *HD-ZIP*, *HSF*, *SCL*, *ARR*, and *SPL*) have been shown to be activated in tea plants in response to cold and drought (Chen et al., 2014; Cao et al., 2015; Zheng et al., 2015; Wang et al., 2016; Cui et al., 2018; Li et al., 2019; Ma et al., 2019; Zhang et al. 2019a). Additionally, signal transduction pathways are the link between the sensing mechanism and the genetic response, and plants can have multiple stress perception and signal transduction pathways, which may cross-talk at various steps in the pathways (Huang et al., 2012). The pathway analysis indicated that “plant hormone signal transduction,” “starch and sucrose metabolism,” “peroxisomes,” and “photosynthesis” might play a vital role in low temperature responses in tree crops (Zhou et al., 2021).

Due to out-breeding and its long gestation period, the tea plant requires next-generation breeding strategies to improve its drought and cold tolerance through a deeper understanding of key regulators and their variants for precision introgressions to have better yield and quality under stress conditions. Therefore, efforts are needed to elucidate the global transcriptomic dynamics of multiple tea genotypes under drought and cold stress to critically discern key molecular players (Parmar et al., 2019). The North Caucasus’ tea germplasm collection is in the border region (44°36′40″ N, 40°06′40″ E) of the possible global tea production and can be the source of the most tolerant cultivars; some genotypes of the collection can survive below −20°C, providing a good yield (Samarina et al., 2022). Resequencing of new germplasm outside of the global producing regions can help to discover novel molecular mechanisms of acclimation and domestication of tree crops in the extreme climatic zones. The third-generation sequencing technology represented by PacBio has the advantage of long read lengths (Sun et al., 2020a). PacBio and RNA-seq sequencing technologies are highly complementary to each other. To obtain an overall view of the molecular regulation during cold, freezing, drought, and recovery, we combined PacBio genome sequencing and RNA-Seq to investigate the transcriptome dynamics of *C. sinensis* cv. Kolkhida. This study will provide new data on overlapping and specific regulatory pathways, key functional genes, and signal transduction components involved in short- and long-term cold, freezing, drought, and recovery in perennial tree crops.

## 2 Materials and methods

### 2.1 Plant material and stress induction

Three-year-old plants of the elite local tea cultivar Kolkhida (the survival temperature of adult plants is about −10–12°C) obtained by vegetative propagation at the Federal Research Centre, the Subtropical Scientific Centre of the Russian Academy of Sciences (FRC SSC RAS, Sochi, Russia), were used for experiments. Healthy plants grown in 2-liter pots filled with brown forest acidic soil (pH = 5.0) were randomly selected for experiments; 9–15 plants per treatment were replicated three times. The plants were maintained for one month at the following control conditions: temperature +22 ± 2°C, light regime day/night 16/8, light intensity of 3,000 lux, soil water content of 65 ± 5%. To induce cold stress, plants were placed in the cold chamber HF-506 (Liebherr, Denmark) at a temperature of +4 ± 2°C for 14 days. Freezing was induced after 14 days of cold by the following decrease of the temperature by −4°C for 7 days. Recovery was induced by a gradual increase in temperature +10 ± 2°C for 10 days. To induce drought stress, the soil watering was gradually decreased to 15% within 14 days, with a 7-day recovery period. The light regime was day/night 16/8, the light intensity was 3,000 lux, and the soil water content was 65 ± 5 for all treatments (**Table 1**).

**Table 1:** Experimental treatments.

Treatment	Temperature, °C	Light/dark	Soil water content, %	Duration,Days
**Control**	+22 ± 2	16/8—3,000 lux	65 ± 5	30
**Cold12h (12 h)**	+4 ± 2	16/8—3,000 lux	65 ± 5	0.5
**Cold3d (three days)**	+4 ± 2	16/8—3,000 lux	65 ± 5	3
**Cold14d (14 days)**	+4 ± 2	16/8—3,000 lux	65 ± 5	14
Freezing3d (three days)*	−4 ± 2	16/8—3,000 lux	65 ± 5	3
Freezing7d (seven days)*	−4 ± 2	16/8—3,000 lux	65 ± 5	7
**RecoveryC (recovery after freezing)**	+10 ± 2	16/8—3,000 lux	65 ± 5	10
**Drought3d (three days)**	+22 ± 2	16/8—3,000 lux	45 ± 5	3
**Drought9d (nine days)**	+22 ± 2	16/8—3,000 lux	25 ± 5	9
**Drought14d (14 days)**	+22 ± 2	16/8—3,000 lux	15 ± 5	14
**RecoveryD (recovery after drought)**	+22 ± 2	16/8—3,000 lux	65 ± 5	7

*Freezing was followed by a 14-day cold.

### 2.2 Phenotypical evaluation of tea plants under stress

For each assessed parameter (relative electrolyte leakage, relative water content, caffeine, theanine, and catechin contents), 2^nd^, 3^rd^, and 4^th^ mature leaves from the top of the plant were used for sampling.

Electrolyte leakage indicating the damage of leaf tissues was measured using the conductivity meter ST300C (Ohaus): a leaf sample was immersed in 150 ml of deionized water, and electrical conductivity was evaluated 2 h later (L1) and after 2 h of boiling and cooling (L2). The relative electrolyte leakage (EL, %) was calculated as: $EL=\frac{L1}{L2}*100$ (Bajji et al., 2002).

The relative water content (RWC) was determined as follows: fresh leaves were first weighed (FW), then immersed in water solution for 12 h (TW) and dried at 105°C for 5 h (DW). RWC was calculated according to the formula: $RWC=\left(\frac{\left(FW-DW\right)}{\left(TW-DW\right)}\right)*100\%$ (Yamasaki and Dillenburg, 1999).

Caffeine, L-theanine, and catechins (gallocatechin (GC), epigallocatechin (EGC), epicatechin (EC), epicatechin gallate (ECG), gallocatechin gallate (GCG), and epigallocatechin gallate (EGCG)) (mg g^−1^ dry leaf mass) were evaluated by HPLC using the following extraction protocol: 130–175 mg of dried tea leaf were poured into a 4.0-ml solution of 80% methanol, hermetically closed, and incubated for one week at +4°С in the dark. After that, the vessels with methanol leaf extracts were placed in a UV bath for 30 min and then centrifuged at 13,000 rpm for 10 min. Then, 1 ml of supernatant was injected into the HPLC column. The Agilent Technologies 1100 HPLC chromatographer, equipped with a flow-through vacuum degasser G1379А, a 4th-channel low pressure gradient channel pump G13111А, an automatic injector G1313А, a column thermostat G13116А, and a diode array detector G1316A, was used. The 2.1 × 150 mm column filled with octadecyl silyl sorbent, with agrain size of 3.5 µm, “ZORBAX-XDB C18,” was applied. The acetonitrile solution was used for the gradient: the initial composition of the mobile phase, consisting of 90% (v/v) solvent A (0.1% H_3_PO4) and 10% of solvent B (90.0% acetonitrile), was maintained for 8 min. After that, solvent A was decreased linearly to 40% at 25 min, 0% at 90 min, and then increased to 100% at 29.1 min to 34 min. The programming was then continued in isocratic mode as follows: 40% A at 70.1 to 75.0 min; 7% A at 75.1 to 90.1 min (the flow rate is 0.30 ml/min, the column temperature is 40°С). The wavelengths for detection were 195 nm (for L-theanine) and 273 nm for caffeine and catechins. Identification of the substances was performed based on the time of holding the standards for respective compounds.

### 2.3 Pac-Bio genome sequencing, assembly, and annotation of cv. Kolkhida

For the PacBio Hifi sequencing, the 20 kb libraries with the three cells of data were constructed following PacBio’s standard protocol and sequenced using the Sequel II platform. For the short reads, DNA samples were sequenced using the Illumina Hiseq platform with 150-bp pair-end reads and an insert size of 350 bp. All the raw sequencing reads were preprocessed to remove low-quality bases, adaptor reads, duplications, and potential contaminants before subsequent analyses. A total of 88.62 Gb (~29-fold) of high-fidelity PacBio reads were sequenced for the Kolkhida tea genome. We assembled reads by Hifiasm (Cheng et al., 2021), and because of the high heterozygosity and repeat sequence number in the tea plant genome, redundant contigs were conducted by Purge_Dups (Guan et al., 2020). To correct the SNP errors and small indel variations, the Pilon tool (Walker et al., 2014) was employed to polish the genome using Illumina short reads. Finally, the assembled genome size was 3.11 Gb with a contig N50 of 4.32 Mb. The BUSCO (Manni et al., 2021) assessment showed 94.3% completeness of core-orthologous genes. Otherwise, the Illumina short reads were mapped to the genome with a 97.57% mapping ratio.

### 2.4 RNA-sequencing and data analysis

The leaf sampling for the RNA extraction was performed between 10 and 12 am (except for the treatment Cold12h, where it was performed at 10 pm). A total of 48 RNA libraries were constructed for sequencing (three to five biological replicates per treatment, each replicate representing a separate plant) using standard Illumina protocols, and RNA sequencing was performed by Novogene Co., Ltd. (https://en.novogene.com/) using the Illumina Hi-Seq platform. Read quality control was performed using fastp (https://github.com/OpenGene/fastp). The RNA-Seq reads were aligned to the reference genome using the HIS AT2 short read aligner tool (Kim et al., 2019). Intermediate file processing of.sam to sorted.bam conversion was carried out using the SAMTOOLS v.1.9 package (Danecek et al., 2021). Based on the alignment BAM file, the RSEM (https://github.com/deweylab/RSEM) (Li and Dewey, 2011) was employed to calculate and quantify the gene expression using the default parameters. Gene expression was normalized by reads per kilobase of exon per million reads mapped. The differentially expressed genes (DEGs) for each of the compared sample groups were identified by the DEseq2 package (Love et al., 2014) with a P-value<0.05, and those DEGs with |log2FC|>1, FDR<0.1 were defined as having significantly different expression. The Venn diagrams of the DEGs among the compared groups were created by the Venn web tool (https://bioinformatics.psb.ugent.be/webtools/Venn/). GO (http://www.geneontology.org/) and KEGG (https://www.kegg.jp/) enrichment of the DEGs was conducted by the Clusterprofiler (https://bioconductor.org/packages/clusterProfiler/).

### 2.5 Gene prediction

We combined *de novo*, homology-based, and RNA-seq methods to predict protein-coding genes in the tea plant cv. Kolkhida genome. AUGUSTUS (Stanke et al., 2006) and GENESCAN (Burge and Karlin, 1997) were conducted for *de novo* prediction. First, the TE sequences were masked from the genome. For the AUGUSTUS, we randomly selected 2,000 high quality genes from the homology-based results as the training sets. Arabidopsis parameters were used for Genescan.

For the homology prediction, the proteins derived from *Actinidia chinensis*, *Arabidopsis thaliana*, *Camellia oleifera*, *C. sinensis* cv. TGY, *Vitis vinifera*, and uniprot_sprot_plants were used as the inputs for TBLASTN (Altschul et al., 1997) with an E-value threshold of 1e−06. Then the blast hits were linked to candidate gene loci by solar (v.0.9.6) (Li et al., 2010) with parameters “-a prot2genome2 –z”. Finally, the Genewise tool (Birney et al., 2004) was employed to construct the intron-exon boundary. Genes with a length of less than 150 bp or with incomplete structures were removed.

For the RNAseq prediction, all the transcript sample data were mapped to the genome using STAR (2.7.9a) (Dobin et al., 2013) and then the alignments were assembled by String (v2.1.7) (Kovaka et al., 2019) with the following parameters: “-j 2 -f 0.01 -c 2 -m 200 -a 10”.

Finally, all pieces of evidence resulting from the above three methods were integrated by the Maker tool (v.01.03v 3.01.03) Pipeline (Cantarel et al., 2008), which can integrate predictions based on homology proteins, *de novo* gene models, and transcriptome assembly genes to better annotate protein-coding genes.

### 2.6 Verification of the RNA-Seq results by qRT-PCR

Total RNA was extracted from the third leaf from the top of the plant in three biological replicates by the Trizol method, according to the manufacturer’s protocol (Biolabmix, Russia; https://biolabmix.ru/). The concentration and quality of RNA were evaluated using a Bio-drop µLite (Biochrom, UK) spectrophotometer, and quality was assessed in a 1% agarose gel. The 1,000 ng RNA was treated with 1 µl DNAseI-Buffer and 1 µl DNAseI (Biolabmix, Russia; https://biolabmix.ru/) for 30 min at 37°C, with subsequent DNAse inhibition by heating to 65°C for 10 min. After that, 1,000 ng of RNA was used to prepare 20 µl of cDNA using the M-MuLV–RH-kit (Biolabmix, Russia; https://biolabmix.ru/), with the following quality evaluation by gel electrophoresis and qRT-PCR using LightCycler 96 (Roche Life Sciences; https://lifescience.roche.com/global_en.html). After cDNA preparation, all samples were diluted to the same concentration of 700 ng µl^−1^ according to the standardization achieved by the expression of the reference gene Actin (NCBI Gene ID: 114316878). To analyze gene expression, the 15 µl of the qPCR-mix consisted of 7.5 µl of 2× SybrBlue qRT-PCR-buffer with the hot-start polymerase (Biolabmix, Russia; https://biolabmix.ru/), 0.2 µl of each primer (forward and reverse), 1 µl cDNA, and the rest volume was PCR-water. The two-step amplification program was applied: preheating for 5 min at 95°C, 40 cycles of amplification (10 s at 95°C, 30 s at 56–62°C), final elongation for 5 min at 72°C, and melting for 3 min at 95°C. The relative gene expression level was calculated by the statistical method of Livak and Schmittgen (2001) using the following algorithm: 2^−ΔΔCq^, where:

ΔΔCq = (Cq_gene of interest_ − Cq_internal control_)_treatment_ − (Cq_gene of interest_ − Cq_internal control_)_control._

### 2.7 Data analysis and availability

Statistical analyses of the data were carried out using XLSTAT software (free trial version) (https://www.xlstat.com/). A one-way ANOVA and Tukey's HSD were performed to determine significant differences between the respective treatments. Additionally, hierarchical clustering was performed, and dissimilarities were calculated using the DICE coefficient, with agglomeration by Ward’s method (Ward 1963). The data were analyzed by Pearson’s (n) PCA type. The co-expression analysis was performed by pairwise Pearson correlation coefficients using the function pandas.DataFrame.corr (https://pandas.pydata.org/docs/reference/api/pandas.DataFrame.corr.html). For calculations, we used log2-values of expression changes in response to cold, freezing, and drought. Pairs of co-expressed genes with a pairwise Pearson coefficient >0.94 were selected for the gene network reconstruction. The table with co-expression above the threshold was imported into Cytoscape v. 3.9.1 for further layout and visualization (Shannon et al., 2003). Additionally, the information about homologs in *A. thaliana* was added to the gene network (**Supplementary Tables S4, S5**).

Raw data from the PacBio HiFi reads and the Illumina short reads, as well as genome assemblies and annotation proteins (gff and fasta) have been deposited in the China National GeneBank Database (project accession number: CNP0004163).

## 3 Results

### 3.1 Phenotypical evaluation of stress effect

**Figure 1:**
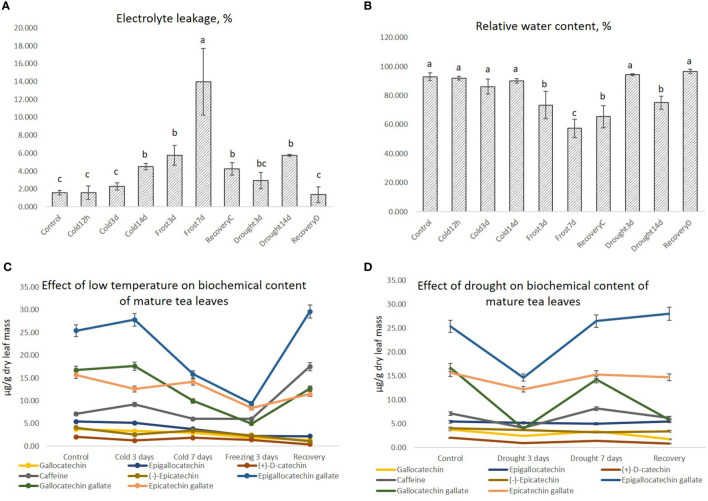
The relative electrolyte leakage **(A)**, the relative water content **(B)**, and the catechins and caffeine content **(C, D)** in the tea leaf tissues under cold, freezing, and drought treatments. Small letters represent the significance of the differences at P<0.05 as compared to the control.

Cold resulted in increased electrolyte leakage (EL) in five of the nine treatments as compared to control, namely Cold14d, Freezing3d, Freezing7d, Drought14d, and RecoveryC (see **Table 1** above for abbreviation decoding). The highest EL (14.0%) was detected under Freezing7d as compared to Control (1.6%). Three other treatments (Cold14d, Freezing3d, and Drought14d) showed about a 3-fold elevation in EL, indicating equal tissue damage in these treatments (**Figure 1A**). The remarkable decrease in RWC was observed in four treatments (Freezing3d, Freezing14d, RecoveryC, and Drought14d)—57.4%–75.1% as compared to Control, which was at 93.0%. Interestingly, no significant decrease in RWC was detected in the other treatments (**Figure 1B**). Additionally, RecoveryC showed lower EL and lower RWC as compared to RecoveryD, indicating delayed recovery after freezing as compared to drought.

Under the effects of cold and freezing, the contents of several catechins, namely EGC, GCG, and EGCG, decreased by 30% compared to the control (**Figure 1C**). The long-term drought has not significantly affected tea quality; however, the short-term drought resulted in a decrease in several catechin contents (EGC, GCG, and EGCG) in tea leaves (**Figure 1D**). Additionally, no significant changes in GC, EGC, and EC were detected among different drought treatments.

### 3.2 Transcriptome dynamics of tea plants under cold, freezing, drought, and recovery

#### 3.2.1 Biological processes and differentially expressed genes under cold, freezing, drought, and recovery

Among the different treatments, the highest number of DEGs was observed under Freezing7d (7915) and Cold14d (7896), with 3,780 and 3,532 genes upregulated, respectively. The lowest number of DEGs was observed under Drought3d (47) and Drought9d (220), with five and 112 genes upregulated, respectively (**Table 2**; **Supplementary Tables S1**, **S2**). The RecoveryC samples indicated about 6.5 times greater DEG numbers as compared to RecoveryD.

**Table 2:** The number of differentially expressed genes in each experimental group.

VS	Upeegulated	Downregulated	Total
Control-VS-Cold12h.DEseq2	701	554	1,255
Control-VS-Cold03d.DEseq2	1,627	1,789	3,416
Control-VS-Cold14d.DEseq2	3,532	4,364	7,896
Control-VS-Frost03d.DEseq2	1,034	2,009	3,043
Control-VS-Frost07d.DEseq2	3,780	41,35	7,915
Control-VS-CRecovey.DEseq2	1,727	1,929	3,656
Control-VS-Drougth03d.DEseq2	5	42	47
Control-VS-Drougth09d.DEseq2	112	108	220
Control-VS-Drougth14d.DEseq2	1,258	667	1,925
Control-VS-DRecovey.DEseq2	130	434	564

**Figure 2:**
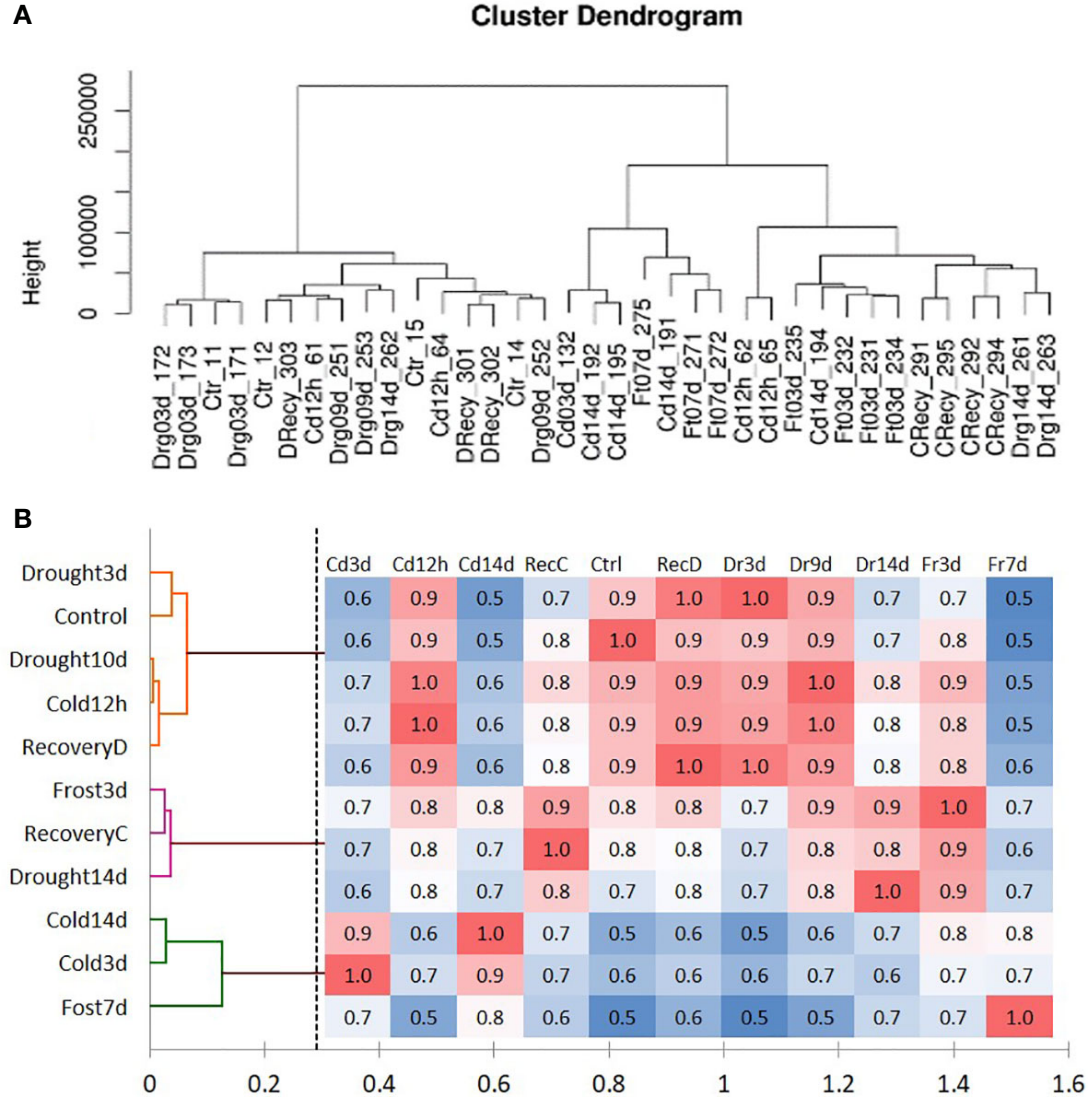
**(A)** UPGMA-clustering of tea transcriptome samples; **(B)** correlation heat map of tea plant stress responses based on transcriptome profiles.

Based on the DEGs dissimilarity, two main significant clusters were identified: the first cluster includes the following samples—Control, Drought3d, Drought9d, and RecoveryD. The second cluster combined all treatments with a pronounced stress response: Cold14d, Drought14d, Freezing3d, Freezing7d, and RecoveryC (**Figure 2A**). The correlation map corresponds with **Figure 2A**, indicating three groups of correlated treatments: 1). Control, Drought3d, Drought9d, Cold12h, and RecoveryD; 2). Freezing3d, RecoveryC, and Drought14; and 3). Cold14D, Freezing7D, and Cold3d (**Figure 2B**). These results correspond with the physiological parameters, confirming the similar stress severity in tea plants subjected to Cold14D, Freezing3d, and Drought14d. Thus, these three treatments were further used to analyze overlapping and specific stress responses.

**Figure 3:**
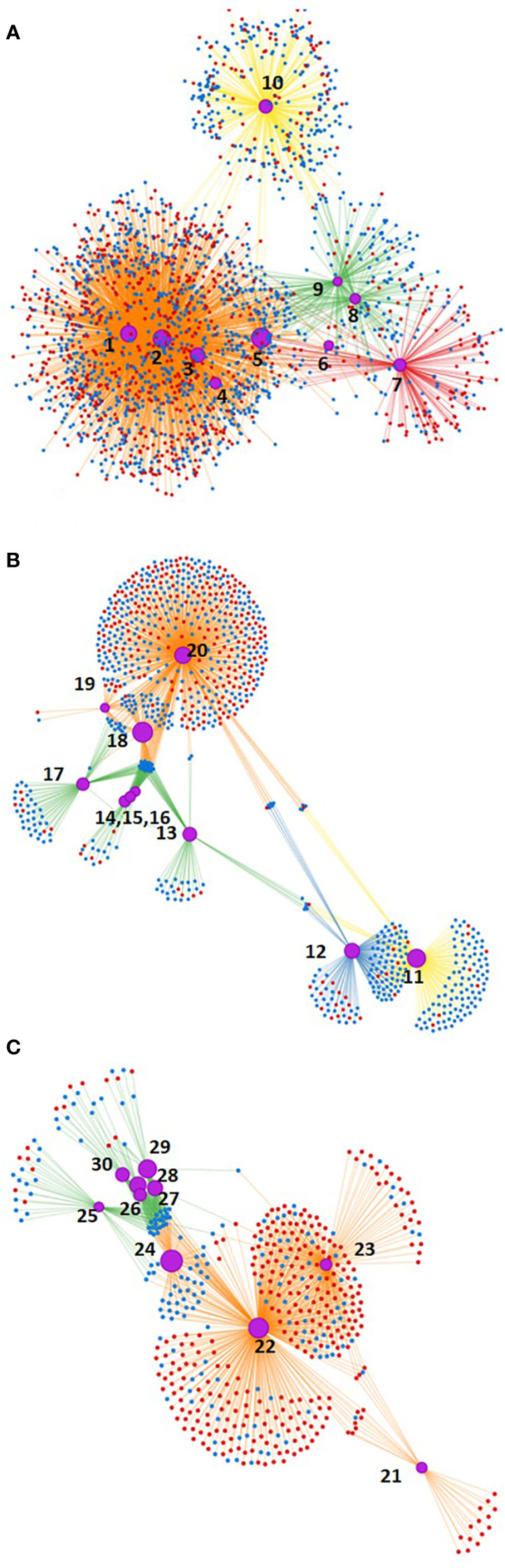
Gene networks affected by Cold14d, Freezing3d, and Drought14d: blue dots—downregulated genes, red dots—upregulated genes, purple cycles marked with numbers—biological processes: **(A)** Cold14d: 1—Biosynthesis of secondary metabolites, 2—Metabolic pathways, 3—carbon metabolism, 4—Glyoxylate and dicarboxylate metabolism, 5—Photosynthesis, 6—Photosynthesis antenna proteins, 7—Endocytosis, 8—RNA-polymerase, 9—Ribosome, 10—Plant–pathogen interaction; **(B)** Freezing3d: 11—Plant–pathogen interaction, 12—MAPK-signaling pathway, 13—RNA-polymerase, 14—Base excision repair, 15—DNA-replication, 16—Mismatch repair, 17—Ribosome, 18—Photosynthesis, 19—Oxidative phosphorylation, 20—Metabolic pathways; **(C)** Drought14d: 21—Galactose metabolism, 22—Metabolic pathways, 23—Biosynthesis of secondary metabolites, 24—Photosynthesis, 25—Ribosome, 26—Nucleotide excision repair, 27—Base excision repair, 28—Mismatch repair, 29—DNA-replication, 30—RNA-polymerase.

Analyzing the gene networks, a more pronounced response was observed under Cold14d as compared to Freezing3d and Drought14d (**Figure 3**). Interestingly, much more DEGs involved in the biosynthesis of the secondary metabolites, carbon metabolism, glyoxylate, and dicarboxylate metabolism were observed under Cold14d, than under Freezing3d and Drought14d.

As for common responses, most of the genes involved in photosynthesis and ribosome assembly were significantly downregulated under Drought14d, Cold14d, and Freezing3d. Most DEGs related to RNA polymerase, plant–pathogen interaction, and metabolic pathways were downregulated under Cold14d and Freezing3d. Many genes involved in the biosynthesis of secondary metabolites were upregulated under Drought14d and Cold14d. As for specific responses, most DEGs related to the MAPK-signaling pathway were specifically downregulated under Freezing3d. However, the big portion of DEGs related to nucleotide excision repair, base excision repair, mismatch repair, DNA replication, and RNA polymerase activity was specifically downregulated under Drought14d. Additionally, many DEGs related to galactose metabolism and metabolic pathways were specifically upregulated under Drought14d.

**Figure 4:**
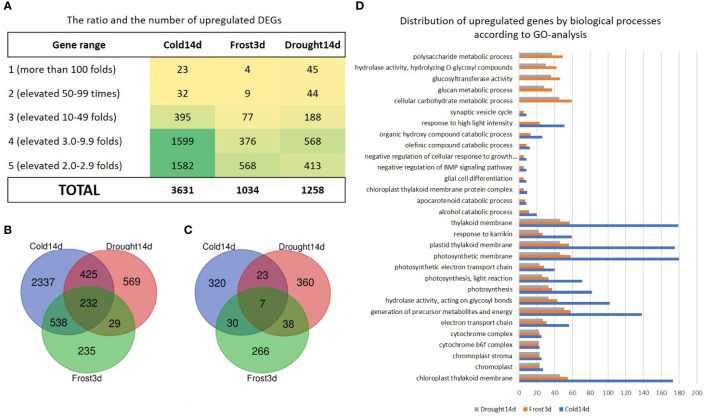
The classification of the significantly upregulated genes under Cold14d, Freezing3d, and Drought14d: **(A)** the ratio and the number of upregulated DEGs, **(B)** the number of common and specific DEGs, **(C, D)** the number of common and specific DEGs related to certain biological processes.

Subsequently, we classified all upregulated genes into five categories by the fold of elevation to control, where I—genes were upregulated greater than 100 folds and V—genes were upregulated up to 2.99 folds (**Figure 4A**). Interestingly, 45, 4, and 23 genes from category I were induced by Drought14d, Cold14d, and Freezing3d, respectively. However, three to four times greater numbers of genes from categories IV and V were observed in Cold14d as compared to Drought14d and Freezing3d. Among all upregulated DEGs, 12%, 10%, and 5% were overlapping for Cold-Freezing, Cold-Drought, and Cold-Freezing-Drought, respectively (**Figure 4B**). Only 1% of genes were common for drought and freezing. Remarkably, 72% of genes were specifically upregulated by each treatment. Additionally, many DEGs of certain biological processes were specifically induced by Drought14d, Freezing3D, and Cold14d (**Figures 4C, D**). For upregulated DEGs, GO terms related to stress and stimulus responses were significantly enriched at all time points. Pathway analysis indicated overlapping GO terms related to thylakoid membranes, photosynthetic membranes, the response to Karrikin, electron transport, and the cytochrome complex play a vital role in tea responses (**Figure 4D**). Interestingly, GO-term enrichment analysis indicates more specific biological processes detected in the tea drought response as compared to the cold and freezing responses.

Among specifically upregulated genes, 57 genes of categories I and II (dramatically upregulated), were specifically induced by Drought14d (**Supplementary Table S3**).

#### 3.2.2 Classification of commonly upregulated DEGs under cold, freezing, and drought

**Figure 5:**
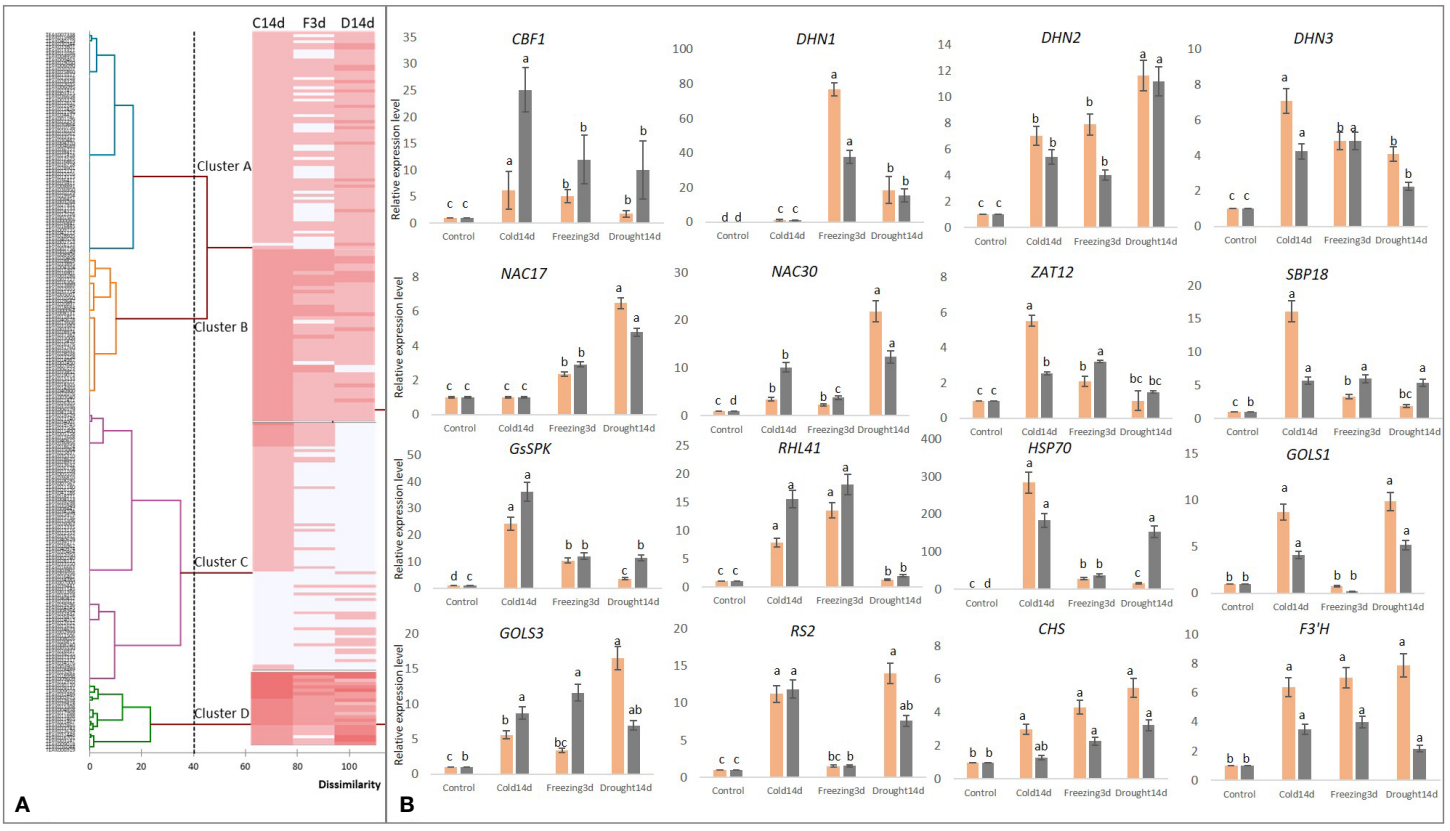
Overlapping genes significantly upregulated in tea plants under Cold14d, Freezing3d, and Drought14d as compared to Control: **(A)** the UPGMAdendrogram and the heat-map based on the level of upregulation according to RNAseq data (see **Supplementary Table S3** for details); **(B)** the relative expression levels of the several stress-inducible genes assessed by qRT-PCR (gray columns) and RNAseq (orange columns). Small letters represent the significance of the differences at P<0.05 as compared to control.

In total, 6.3% of overlapping (common) upregulated genes present in 232 genes and appeared to be the core Cold-Drought-Freezing-responsive genes. These genes clustered into four groups (Clusters A, B, C, and D) according to their expression level (**Figure 5**; **Supplementary Table S3**).

The group D combined 22 genes with expression increased by 50–100-fold as compared to control, namely, *ELIP1*, *ECP63-like*, *GRF1*, *UGT74B1*, *glucan endo-1,3-beta-glucosidase 11-like*, *12-like*, *phosphoprotein ECPP44*, *sugar transport protein 13-like*, *protein NRT1/PTR FAMILY 1.2-like*, *F-box/kelch-repeat protein SKIP25-like, ultraviolet-B receptor UVR8-like, 11S globulin seed storage protein 2-like*, *EID1-like F-box protein 3*, *=probable protein phosphatase 2C 24*, and seven more that showed no overlap with any annotated genes and can represent novel protein-coding genes). Similarly, the expression groups B and C combined 71 and 46 genes, respectively, which were upregulated up to 49 folds. Additionally, the most abundant expression group A combined 93 genes with a moderate increase of up to 9 folds. Along with well-known regulators of stress response (e.g., *CBF* and *DHN*), we identified novel transcripts that were highly induced by cold, freezing, and drought (e.g., *ELIP1* and *GRF1-interacting factor 1*).

Interestingly, most of the genes in categories IV and V were related to the same category in Cold14d, Freezing3d, and Drought14d, indicating their similar role and co-expression character in the three stress treatments. In addition, transcription factors and metabolism-related genes were observed in each of the expression groups from 1 to 5. Among the 1,492 transcription factor DEGs identified in all treatments, 20 were common in long-term cold, freezing, and drought (**Supplementary Table S3**).

These 20 transcription factor genes correspond to *CBF1*, *DHN1*, *DHN2*, *DHN3*, *LEA*, *LEA14-A*, *LEA29*, *bHLH*, *WRKY22*, *ZAT10*, *NAC*, *GIF1*, *AP2*, *ERF*, *PIP7a*, *GRAS*, *ECP63*, *HSP70*, *DUF567*, and *COR413PM1-like*, and most of them are known to be related to the ABA-signaling pathway (**Supplementary Table S3**).

Remarkably, 36 genes related to hormone-, calcium-, and ROS-signaling were revealed among these 232 DEGs. Additionally, 26 genes were related to cell wall remodeling and biosynthesis: 19 DEGs were related to lipid metabolism, 12 DEGs were related to the biosynthesis of polyphenols and anthocyanidins, 11 DEGs were involved in sugar metabolism and transport, nine DEGs were related to photosynthetic activity and stomatal organization, and eight DEGs were related to amino acid biosynthesis and transport. Also, several genes related to signaling pathways, cell wall remodeling and biosynthesis, and amino acid biosynthesis and transport were not functionally annotated. The rest part of upregulated DEGs were related to protein ubiquitination (*UBC24*, *PUB1*, *PUB19*, *PUB14, PUB45*, *SKIP25*, *NPL4*, *ALT13*, and *ATL51*) and protein catabolism (*CBP2*, *TIC20-V*, *RPN7*, *APF2*, and *ASPG1*), inorganic ion transport (*NRT2.7*, *NRT1*, *ZIP6*, *ZIP8*, *BOR5*, *ACA1*, *FER3*, *FRO2*, *At1g07590*-like, and two Potassium ion transporters), ABC-transport (*ABCC5*, *ABCC3*, *ABCC13*, *ABCC8*, and *ABCF1*), post transcriptional regulation of gene expression (*SNRPB*, *RCL*, *IWC1*, *CDA1*, and *URT1*), DNA-methylation and chromatin modification (*H3.3*, *H2B.1*, and *PELP1*), and NAD-metabolism (*NDA1*, *NADSYN*, and *GID8*) (**Supplementary Table S3**).

Interestingly, 27 novel uncharacterized transcripts were found among these 232 overlapping DEGs, which were highly induced by cold, freezing, and drought and were not recognized and not annotated by publicly available databases, namely TEAK032489, TEAK033550, TEAK006579, TEAK006919, TEAK029131, TEAK010562, TEAK012137, TEAK013321, TEAK015517, TEAK016462, TEAK036810, etc. (**Supplementary Table S3**).

To validate the expression data obtained by RNA-Seq, we performed qRT-PCR analysis of the expression of the set of stress-inducible genes under long-term cold, freezing, and drought (**Figure 5**). In general, both data sets were congruent. The important regulators of the stress responses in tea showed 10-fold elevated expression during long stress treatments, namely *CBF1*, *DHN1*, *DHN2*, *DHN3*, *ZAT12*, *GS-SPK*, *SBP18*, and *HSP70*. Several stress-inducible sugar-metabolism genes (*GOLS1*, *GOLS3*, and *RS2*) were upregulated up to 10-fold. Two important polyphenol biosynthesis transcripts (*CHS* and *F3’H*) were upregulated 2–8 fold in all treatments as compared to control, which is consistent with RNASeq data. However, GOLS1 and RS2 indicated inhibited expression under freezing.

**Figure 6:**
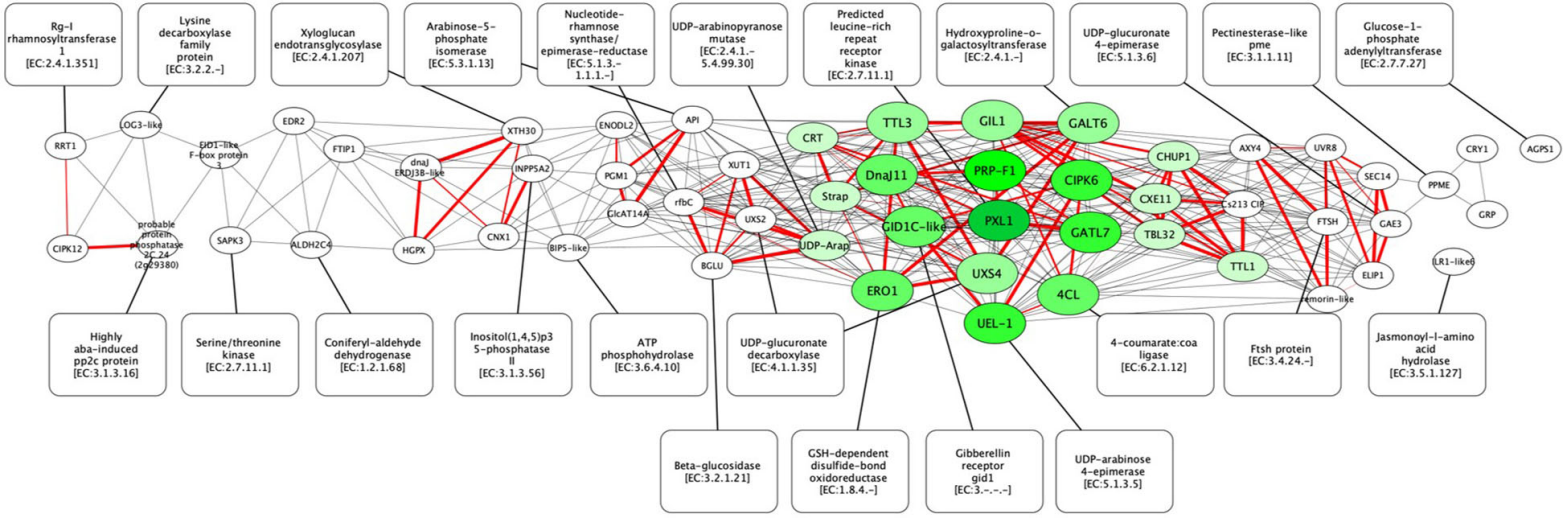
Homology based gene co-expression network for *C. sinensis* (N nodes = 58; N edges = 390). Genes are presented as ellipses, and their corresponding enzymes are presented in round rectangles with an EC number. Genes with the greatest number of edges are presented by shades of green (a color legend is shown on the left bottom part of the figure). The thick red line shows the most significant co-expression interactions (Pearson coefficient >0.997).

To summarize overlapping stress responses and to propose new candidate genes for molecular breeding, we performed co-expression analysis and network reconstruction of the 58 key upregulated DEGs related to signal transduction, light perception, and cell wall remodeling. In total, 390 highly correlated pairs (Pearson coefficient >0.94) were revealed for 54 genes. The KEGG annotations of homologs in *A. thaliana* provided the co-expression gene network with corresponding enzyme annotations (**Supplementary Table S5**). This network contains 56 genes and 415 edges, of which 390 are co-expressed and 25 are enzyme associations (**Figure 6**). Among them, the central functional cluster of 19 genes was revealed with the highest co-expression connectivity (green color). Among these 19 genes, seven are related to cell wall remodeling (*GATL7*, *UXS4*, *PRP-F1*, *4CL*, *UEL-1*, *UDP-Arap*, and *TBL32*), four are related to Ca^2+^-signaling (*PXL1*, *Strap*, *CRT*, and *CIPK6*), three are related to photo-perception (*GIL1*, *CHUP1*, and *DnaJ11*), two are related to hormone signaling (*TTL3* and *GID1C-like*), two are related to ROS signaling (*ERO1* and *CXE11*), and one is related to the phenylpropanoid pathway (*GALT6*). This cluster also contains 41 of 79 most significant edges (Pearson coefficient >0.997) that confirms the joint regulation of this core sub-network. Among them, four genes with six most significant neighbors were identified: *GIL1*, *Strap*, *CRT*, and *CXE11*. Additionally, five genes from this cluster are characterized by the highest number of edges in the network: *PXL1*, *PRP-F1*, *GATL7*, *CIPK6*, and *UEL-1* (**Supplementary Table S6**).

## 4 Discussion

Tea genetic diversity in the North-Western Caucasus can be considered a useful reservoir of cold-tolerant germplasm useful for the development of new tolerant cultivars. Kolkhida is the best large-leaf local tea cultivar, characterized by high leaf quality for black and green tea production. In this study, we analyzed the transcriptome dynamics of this cultivar in response to the cold, freezing, drought, and recovery. Many previous studies focused on the short-term stress response. However, the long-term response and recovery mechanisms can be of critical importance for crop hardening. In this study, the emphasis was placed on overlapping genes and pathways because common mechanisms are important to develop new cultivars tolerant to several stresses and showing higher hardening potential. We identified a series of new candidate genes related to different pathways. These genes can be used as new genetic targets for gene editing and marker-assisted selection.

Among the different treatments, the highest number of DEGs was observed under freezing and cold conditions rather than drought (**Table 2**). In addition, the RecoveryC samples indicated about 6.5 times greater DEG numbers as compared to RecoveryD. These results correspond with several studies that reported that cold induced more DEGs than drought in tea plants (Zheng et al., 2015), apples (Li et al., 2019), maize (Lu et al., 2017), and tomatoes (Zhou et al., 2019). Furthermore, we observed that only 6.3% of upregulated genes were common for Cold14d, Freezing3d, and Drought14d, whereas 7.9% of upregulated DEGs were common for cold and drought. Other studies showed that only 10% of the drought-inducible genes were also induced by cold in Arabidopsis and tomato (Seki et al., 2002; Zhou et al., 2019). However, these researchers evaluated the short-term responses while we focused on the long-term response. Anyway, the percentage of the common upregulated DEGs is not as high as it was expected, and most of the DEGs were specific to a cold or drought response.

### 4.1 Overlapping biological processes upon cold, freezing, and drought

Our results on pathway analysis are partly consistent with the other studies, which indicated common GO terms of “thylakoid membranes,” “photosynthetic membranes,” “response to Karrikin,” “electron transport,” and “cytochrome complex” (Zhu et al., 2013; Guo et al., 2021; Zhou et al., 2021). We speculate that these pathways play a vital role in tea responses to long-term drought, cold, and freezing. According to some other studies, “plant hormone signal transduction,” “starch and sucrose metabolism,” “peroxisomes,” and “photosynthesis” might play a vital role in wild apple responses to freezing stress (Zhou et al., 2021). Additionally, Zheng et al. (2015) reported that “photosynthesis” and “photosynthesis-antenna proteins” were important for the short-term cold response. In cotton, treated with 10 d of drought and 2 d chilling of stress, most of the DEGs were related to “carbohydrate metabolism,” “stress/defense response,” “nucleic acid metabolism,” or “transcriptional regulation” (Zhu et al., 2013), which is consistent with our results on the tea plant. Long-term downregulation of many photosynthesis genes can involve different mechanisms in drought and cold (Hussain et al., 2018). The difference in GO results can be explained by the different lengths of stress treatments used in the studies.

#### 4.1.1 Phenylpropanoid pathway and cell wall remodeling DEGs

Among common upregulated genes, several key genes were related to the phenylpropanoid pathway (*ALDH2C4* and homolog *At1g55270*—regulator of the phenylpropanoid pathway) and the polyphenol biosynthesis pathway (*F3’H* (two genes), *F3’5’H* (two genes), and *CHS1*). Interestingly, along with the increased expression of *CHS1*, *F3’H*, and *F3’5’H*, we observed inhibited expression of their downstream genes (*DFR* and *FLS*), and these results corresponded with the HPLC results (**Figure 1C**). Thus, it can be suggested that the conversion of dehydroflavonols into the final products is suppressed under long-term stress. Several other transcriptomic studies indicated upregulation of the polyphenol-related DEGs controlled by *WD40*-*MYB*-*bHLH* regulatory complexes and could be an important mechanism of plant defense against different abiotic stresses (Chezem and Clay, 2016; Xie et al., 2018; Qari and Tarbiyyah, 2021; Wang et al., 2021).

The phenylpropanoid pathway serves as a rich source of metabolites in plants. It is a starting point for the biosynthesis of lignin, flavonoids, and coumarins (Fraser and Chapple, 2011; Hori et al., 2020; Oliveira et al., 2020). We identified many upregulated genes related to cell wall remodeling and biosynthesis (*UDP-Arap*, *XTH30*, *AGPS1*, *BGLU*, *ENODL2*, *AXY4*, *UEL-*1, *PRP-F1*, *API*, *PPME*, *GALT6*, *GATL7*, *UXS2*, *UXS4*, *TBL32*, *GlcAT14A*, *XUT1*, *GAE3*, *4CL*, *API*, *RRT1*, *rfbC*, glucan endo-1,3-beta-glucosidase 7- and 8-like, etc.), confirming the importance of these pathways for the long-term cold, freezing, and drought responses in tea plants. Recent studies showed upregulation of lignin biosynthesis genes along with the downregulation of cellulose biosynthesis genes under several osmotic stresses (Wildhagen et al., 2018; Chen et al., 2019; Hori et al., 2020). Additionally, an increased level of xyloglucan endotransglucosylase/hydrolase (XTH) and expansin proteins were highlighted (Peña et al., 2012; Gall et al., 2015). These proteins affect the cell wall plasticity and reinforcement of the secondary wall with hemicellulose and lignin to increase cell wall thickening. In our study, the increased expression of beta-glucosidase (BGLU) and several DEGs related to xyloglucan and pectin biosynthesis (*RRT1*, *PPME*, *XTH*, *UXS2*, *UXS4*, *GAE3*, and *XUT1*) was observed. Among them, *RRT1* is required for the synthesis of the RG-I major structural domain of pectin, which is important for both cellular adhesion and cell wall plasticity (Takenaka et al., 2018); *PPME*, participates in apoplastic Ca^2+-^homeostasis, controlling stomatal movements, and regulating the flexibility of the guard cell wall (Wu et al., 2018). Also, the products of *XTH*s cut and re-join hemicellulose chains in the plant cell wall, affecting cellulose deposition (Wu et al., 2018). Additionally, there are *UXS2*, *UXS4*, and *GAE3*, required for the biosynthesis of heteroxylans and xyloglucans and for the side chains of pectin (Kuang et al., 2016; Borg et al., 2021).

Plant cell walls contain hydroxyproline-rich O-glycoproteins (HRGPs), a superfamily that is classified into extensins (EXTs), arabinogalactan-proteins (AGPs), and Hyp/Pro-rich proteins (H/PRPs) (Cassab and Varner, 1988; Basu et al., 2015; Ajayi et al., 2021). According to our results, a set of genes involved in H/PRPs and AGPs metabolism (*AGPS1*, *UEL-1*, *API*, *GALT6*, *GATL7*, *GlcAT14A*, *ENODL2*, *PRP-F1*, etc.) were highly upregulated, suggesting that glycosylation of HRGPs is an important responsive mechanism under long-term stress. Additionally, some genes (*TBL32*, *TBL27*) related to O-acetylation of polysaccharides were upregulated under long-term stresses in the tea plant, which is consistent with some earlier findings (Sun et al., 2020b). O-acetylation of polysaccharides changes their physicochemical properties, and acetyl substituents inhibit the enzymatic degradation of wall polymers (Gall et al., 2015).

To summarize, several important mechanisms of long-term stress responses can be suggested that are directed toward increasing the cell wall’s plasticity, thickness, and hydrophobicity: lignin biosynthesis, glycosylation of HRGPs, O-acetylation of polysaccharides, pectin biosynthesis and branching, and xyloglucan and arabinogalactan biosynthesis.

#### 4.1.2 Light-perception- and signal transduction DEGs

According to the KEGG analysis, most of the common biological processes in tea were related to membranes, electron transport, and light perception. Previous studies also reported that cold response is closely associated with light perception, particularly the circadian clock, which affects the expression of *CBF* genes (Chen et al., 2004; Gould et al., 2013; Wisniewski et al., 2014; Estravis-Barcala et al., 2019; Kidokoro et al., 2021; Kidokoro et al., 2022). In accordance with these findings, we revealed increased expression of genes related to red-light perception (*GIL1*), blue and UV-light perception (*CRY1*, *ELIP1*, and *UVR8*), chloroplast relocation (*CHUP1*), regulation of chlorophyll biosynthesis (clone *Cs213* putative cold-inducible protein), stomatal movement (*PGM1*), and PSII-associated light-harvesting complex II (*FTSH*). Interestingly, among all upregulated DEGs, the highest expression level was observed in *ELIP1*, which was upregulated 150–2,000 folds above control under long-term cold, freezing, and drought. Early light-inducible proteins (ELIPs) are present in the thylakoid membranes. These proteins protect photosynthetic machinery from various environmental stresses in higher plants and have been suggested to participate in the phytochrome signaling pathway (Rizza et al., 2011). The induction of *ELIP1*/*2* expression is mediated *via CRY1* in a blue light intensity-dependent manner (Kleine et al., 2007; Yang et al., 2017). *CRY1* participates in the high temperature response in plants. However, an accumulation of *CRY* transcripts has not been observed in response to short-term cold stress in Arabidopsis (Gould et al., 2013; Ma et al., 2016). Several other genes related to the light perception were upregulated under long-term cold, freezing, and drought. Among them, *EID1*-like F-box protein 3 is related to red-light perception and functions as a negative regulator in phytochrome A (phyA)-specific light signaling (Marrocco et al., 2006). *DnaJ11* and *dnaJ ERDJ3B*-like encode co-chaperone components, responsible for stabilizing the interaction of Hsp70 with client proteins (Ohta and Takaiwa, 2014). Knockout of these genes in *A. thaliana* causes a decrease in photosynthetic efficiency and destabilization of PSII complexes (Chen et al., 2010). Also, the FT-interacting protein 3 *FTIP1* is an essential regulator of FT encoding florigen in plants (Liu et al., 2012). To summarize, it can be suggested that the long-term overlapping stress responses include the activation of several important genes of photo-perception, which probably activate the phenylpropanoid pathway leading to cell wall remodeling. An adjustment in the light harvesting system and reaction centers to capture less light energy for photosynthesis can be an important regulatory mechanism for long-term stress, according to Zhou et al. (2021). We suppose that several light receptor genes, such as *CRY1*, *ELIP1*, *FTIP1*, *EID1*, *ERDJ3B*, and *dnaJ11*, can be new target genes for molecular breeding of the tea plant.

*Calcium signaling.* Under the long-term stresses, we observed the elevated expression of several genes related to Ca^2+^-dependent signaling and protein phosphorylation. Among them are *CNX1* and *CRT*, whose products act as molecular chaperones (Liu et al., 2017; Joshi et al., 2019); *CIPK12* and *CIPK6* products bind to CBLs—which are regulators of Ca^2+^-signal transduction (Sardar et al., 2017; Czolpinska and Rurek, 2018; Guo et al. 2018; Bai et al., 2022). Additionally, several genes related to Ca^2+^-signaling were upregulated (Strap, *SAPK3*, *PXL1*, *INPP5A2*, *GRP*, etc.), indicating the important role of the membrane trafficking system in response to long-term cold, freezing, and drought in tea plants. Similarly, in Populus, calcium-dependent protein kinase 10 (*CPK10*) activates both drought- and frost-responsive genes to induce stress tolerance (Chen et al., 2013). In apples, common DEGs encoding protein phosphatases and serine/threonine protein kinases were upregulated in response to different abiotic stresses (Li et al., 2019). In addition, probable protein phosphatase 2C 24 (2g29380) was upregulated in tea plants. PP2C enzymes are key players in plant signal transduction processes such as ABA signal transduction (Rodriguez, 1998). These results confirmed that activation of Ca^2+^-signaling cascades is relevant for the long-term stress responses in tea plants.

*Hormone signaling*. Several new DEGs involved in hormone signaling were upregulated in tea plants under long-term stress (*GID1C*-like, *LOG3*-like, *ILR1*-like6, *TTL1*, *TTL3*, and *2g29380*). The product of *GID1* can bind negative regulators of GA responses called DELLA proteins (Hauvermale et al., 2014). *LOG* plays a pivotal role in regulating cytokinin activity (Kuroha et al., 2009). *ILR1* regulates the rates of amido-IAA hydrolysis, resulting in the activation of auxin signaling (Sanchez Carranza et al., 2016). *TTL1* regulates the transcript levels of several dehydration-responsive genes, such as *CBF2*, *ERD1* (early response to dehydration 1), *ERD3*, and *COR15a* (Rosado et al., 2006; Lakhssassi et al., 2012). Our results indicate that jasmonic acid, brassinosteroid-, and the ABA-signaling pathways are consistently upregulated during long-term stress, which is partly consistent with recent studies on woody plants (Wisniewski et al., 2014; Estravis-Barcala et al., 2019; He et al. 2019; Zheng et al., 2022). It is suggested that a highly variable interaction between different hormone signal transduction pathways takes place, leading to a complex transcriptional landscape in response to abiotic stress.

*ROS signaling.* A well-known effect of abiotic stress in plants is the production of ROS, which can eventually oxidize lipids, proteins, and DNA and thereby trigger cell death (Akula and Ravishankar, 2011; Bartwal et al., 2013; Estravis-Barcala et al., 2019; He et al., 2019). We revealed several new upregulated DEGs related to lipid metabolism. For example, *SEC14* is an important regulator of phospholipid metabolism (de Campos and Schaaf, 2017); *EDR2* is a negative regulator of cell death and acts in opposition to the SA pathway (Vorwerk et al., 2007); *REM* encodes remorin-like proteins of lipid rafts and physically interacts with receptor-like kinases and pathogen effectors (Cai et al., 2020); *HGPX* encodes phospholipid hydroperoxide (glutathione peroxidase), which participates in scavenging of lipid hydroperoxide (Jain and Bhatla, 2014). Also, *ERO1* encodes endoplasmic reticulum oxidoreductin) that participates in protein folding under oxidative stress (Matsusaki et al., 2019). *CXE11* encodes carboxylesterase 11, which is involved in the catabolism of volatile esters and activation of MeJA signaling (Cao et al., 2019). Finally, two luminal-binding protein genes (*BIP5*-like) whose overexpression leads to an increase in anti-oxidative defenses under water stress in transgenic tobacco and soybean (Valente et al., 2009). These results suggest that lipid stabilization can be an important mechanism of long-term stress responses in tea plants.

The co-expression analysis revealed the core functional module of the network (**Figure 6**), which demonstrates the coordinated regulation of pathways for photo-perception, phenylpropanoid, сalcium signaling, сell wall remodeling, ROS, and hormone signaling. Having key knowledge about the presence of such coordinated regulation and its targets opens a new horizon for the discovery of major activators that play a key role in transcriptional stress responses. In comparison to the core gene network of *A. thaliana* in response to high light (Bobrovskikh et al., 2022), our selected gene set includes four corresponding orthologs*: dnaJ ERDJ3B*-like (TEAK028829/AT3G62600), *ELIP1* (TEAK023638/AT3G22840), *CRY1* (TEAK018624/AT4G08920), and *ALDH2C4* (TEAK002753/AT5G42020). However, these genes are not included in the central co-expression cluster, indicating a high specificity of the highlight stress response as well as different genetic mechanisms involved in these responses.

#### 4.1.3 Regulatory mechanisms

Only 19 transcription factor genes were commonly upregulated (*CBF1*, *DHN1*, *DHN2*, *DHN3*, *LEA*, *LEA14-A*, *LEA29*, *ECP63*, *bHLH*, *WRKY22*, *ZAT10*, *NAC*, *AP2*, *ERF*, *GIF1*, *PIP7a*, *HSP70*, *GRAS*, *DUF567*, and *COR413PM1*-like), and most of them are related to the ABA-signaling pathway. Among the well-known genes, we found new COR genes upregulated under long-term cold, freezing, and drought in tea plants. For example, a homolog of *COR413PM1* is a regulator of the ABA response in Arabidopsis, and affects the ABA-induced transient Ca^2+^ oscillation in the plasma membrane (Hu et al., 2021). Additionally, *DUF567* encodes a protein of unknown function (Nabi et al., 2020), *GRAS*, and *GIF1* (GIF1/2/3), which regulate root and shoot development (Tian et al., 2004; Zhang et al., 2018; Liu et al., 2020). Corresponding with our results, cold and drought induce transcription factors of an ABA-dependent response, such as members of the basic-domain leucine zipper (*bZIP*) family, the *MYB* family, and the *WRKY* family (Li et al., 2019; Zhou et al., 2019; Zheng et al., 2022).

### 4.2 Specific biological processes activated by cold, freezing, and drought

In our experiment, freezing treatment was followed a 14-day cold treatment, leading to the accumulation of the negative effects of low-temperature stress. Most of the DEGs related to the MAPK-signaling pathway were specifically downregulated under freezing, indicating severe damage of the defense system. Mitogen-activated protein kinase cascade (MAPK) is an evolutionarily conserved signal transduction module involved in transducing extracellular signals to the nucleus for appropriate cellular adjustment (Sinha et al., 2011). In strawberries, the freezing stress response was through several pathways: flavonoid biosynthesis, plant hormone signal transduction, MAPK-signaling, starch and sucrose metabolism, and circadian rhythm (Zhang et al., 2019b). Transcriptomic analysis of *Magnolia wufengensis* under cold stress showed that the response mechanism was related to photosynthesis, plant hormone signal transduction, and primary and secondary metabolism pathways (Deng et al., 2019).

Several specific biological processes were indicated in the tea plant under long-term drought. Interestingly, under long-term drought, downregulation of many DEGs related to nucleotide excision repair, base excision repair, mismatch repair, DNA replication, and RNA polymerase activity was detected. DNA repair mechanisms are important to eliminate errors in replication and maintain genomic integrity of plants under endogenous and exogenous DNA-damaging factors. Nucleotide excision repair is a general repair mechanism employed by both prokaryotic and eukaryotic cells to remove a variety of structurally different DNA lesions (Manova and Gruszka, 2015). Base excision repair is a critical DNA repair mechanism for the removal of damaged bases arising from oxidation, alkylation, or deamination (Krokan and Bjoras, 2013; Roldán-Arjona et al., 2019). The mismatch repair (MMR) mechanism of correction of replication errors (mismatches) or nucleotides accidentally inserted/deleted during replication to prevent mutation accumulation (Belfield et al., 2018). Thus, downregulation of these genes can lead to the accumulation of mutations in plant cells under long-term drought stress. Additionally, many DEGs related to galactose metabolism and metabolic pathways were specifically upregulated in Drought14d, which is consistent with other studies on osmotic stress (Seifert et al., 2002; Zhang et al., 2015; Zhang et al., 2019b).

## 5 Conclusion

To conclude, only 17.9% of the cold-induced genes were also upregulated by drought, and only 6.3% of upregulated genes were common for long-term cold, freezing, and drought. In total, 1,492 transcription factor genes were identified among all DEGs related to 57 families. However, only 20 transcription factor genes were commonly upregulated by long-term cold, freezing, and drought (*CBF1*, *DHN1*, *DHN2*, *DHN3*, *LEA*, *LEA14-A*, *LEA29*, *ECP63*, *bHLH*, *WRKY22*, *ZAT10*, *NAC*, *AP2*, *ERF*, *GIF1*, *PIP7a*, *HSP70*, *GRAS*, *DUF567*, and *COR413PM1*-like). Coexpression analysis and network reconstruction showed 19 genes with the highest co-expression connectivity: seven genes are related to cell wall remodeling (*GATL7*, *UXS4*, *PRP-F1*, *4CL*, *UEL-1*, *UDP-Arap*, and *TBL32*); four genes are involved in Ca2+-signaling (*PXL1*, *Strap*, *CRT*, and *CIPK6*); three genes are related to photo-perception (*GIL1*, *CHUP1*, and *DnaJ11*); two genes of hormone signaling (*TTL3* and *GID1C-like*); two genes of ROS signaling (*ERO1* and *CXE11*); and one gene of phenylpropanoid pathway (*GALT6*). These genes can be a new target for molecular breeding of tea plants. Several important mechanisms of long-term stress responses include cell wall remodeling through lignin biosynthesis, o-acetylation of polysaccharides, pectin biosynthesis and branching, and xyloglucan and arabinogalactan biosynthesis. These results revealed the important mechanisms of overlapping responses to cold, freezing, and drought stresses and a set of new target candidate genes for the molecular breeding of tea plants aimed at tolerance to abiotic stresses.

## Data availability statement

The datasets presented in this study can be found in online repositories. The names of the repository/repositories and accession number(s) can be found below: https://db.cngb.org/, CNP0004163.

## Author contributions

LS - conceptualization, experimental design, investigation, data analysis, visualization, funding acquisition, manuscript draft; SW - investigation, data analysis, visualization, manuscript review and editing; LM - funding acquisition, data acquisition, manuscript review and editing; AB and AD - data analysis and interpretation, manuscript critical revision; NK, RS and AM - investigation, data acquisition; JF, KM and AF - results interpretation, formal analysis, manuscript review and editing; SL, TT and AR - results acquisition, revision the work, EK - conceptualization, supervision, formal analysis, manuscript review and editing. All authors approved the final version of the manuscript. All authors agreed to be accountable for all aspects of the work in ensuring that questions related to the accuracy or integrity of any part of the work are appropriately investigated and resolved.

## Supplementary material

Supplementary Table 1 RPKM per treatment.

Supplementary Table 2 All DEGs per treatment.

Supplementary Table 3 Upregulated DEGs.

Supplementary Table 4 Correlation of DEGs.

Supplementary Table 5 Enzymatic Fractions of DEGs.

Supplementary Table 6 Gene network details.
